# Preserving Informative Presence: How Missing Data and Imputation Strategies Affect the Performance of an AI-Based Early Warning Score

**DOI:** 10.3390/jcm14072213

**Published:** 2025-03-24

**Authors:** Taeyong Sim, Sangchul Hahn, Kwang-Joon Kim, Eun-Young Cho, Yeeun Jeong, Ji-hyun Kim, Eun-Yeong Ha, In-Cheol Kim, Sun-Hyo Park, Chi-Heum Cho, Gyeong-Im Yu, Hochan Cho, Ki-Byung Lee

**Affiliations:** 1 AITRICS Corp., Seoul 06221, Republic of Korea; tys@aitrics.com (T.S.); steve@aitrics.com (S.H.); kj@aitrics.com (K.-J.K.); ey10.cho@aitrics.com (E.-Y.C.); jerry@aitrics.com (Y.J.); leah@aitrics.com (J.-h.K.); 2 Division of Geriatrics, Department of Internal Medicine, Yonsei University College of Medicine, Seoul 03722, Republic of Korea; 3 Department of Internal Medicine, Keimyung University Dongsan Hospital, Keimyung University School of Medicine, Daegu 42601, Republic of Korearki0411@hanmail.net (G.-I.Y.); 4 Division of Cardiology, Department of Internal Medicine, Keimyung University Dongsan Hospital, Keimyung University School of Medicine, Daegu 42601, Republic of Korea; kimic.cardiologist@gmail.com; 5 Division of Pulmonary and Critical Care Medicine, Department of Internal Medicine, Keimyung University Dongsan Hospital, Keimyung University School of Medicine, Daegu 42601, Republic of Korea; ibagu70@gmail.com; 6 Department of Obstetrics and Gynecology, Keimyung University Dongsan Hospital, Keimyung University School of Medicine, Daegu 42601, Republic of Korea; c0035@dsmc.or.kr; 7 Division of Pulmonary, Allergy and Critical Care Medicine, Department of Internal Medicine, Chuncheon Sacred Heart Hospital, Hallym University Medical Center, Chuncheon 24253, Republic of Korea

**Keywords:** artificial intelligence, early warning score, imputation, national early warning score, modified early warning score

## Abstract

**Background/Objectives:** Data availability can affect the performance of AI-based early warning scores (EWSs). This study evaluated how the extent of missing data and imputation strategies influence the predictive performance of the VitalCare–Major Adverse Event Score (VC-MAES), an AI-based EWS that uses last observation carried forward and normal-value imputation for missing values, to forecast clinical deterioration events, including unplanned ICU transfers, cardiac arrests, or death, up to 6 h in advance. **Methods:** We analyzed real-world data from 6039 patient encounters at Keimyung University Dongsan Hospital, Republic of Korea. Performance was evaluated under three scenarios: (1) using only vital signs and age, treating all other variables as missing; (2) reintroducing a full set of real-world clinical variables; and (3) imputing missing values drawn from a distribution within one standard deviation of the observed mean or using Multiple Imputation by Chained Equations (MICE). **Results:** VC-MAES achieved the area under the receiver operating characteristic curve (AUROC) of 0.896 using only vital signs and age, outperforming traditional EWSs, including the National Early Warning Score (0.797) and the Modified Early Warning Score (0.722). Reintroducing full clinical variables improved the AUROC to 0.918, whereas mean-based imputation or MICE decreased the performance to 0.885 and 0.827, respectively. **Conclusions:** VC-MAES demonstrates robust predictive performance with limited inputs, outperforming traditional EWSs. Incorporating actual clinical data significantly improved accuracy. In contrast, mean-based or MICE imputation yielded poorer results than the default normal-value imputation, potentially due to disregarding the “informative presence” embedded in missing data patterns. These findings underscore the importance of understanding missingness patterns and employing imputation strategies that consider the decision-making context behind data availability to enhance model reliability.

## 1. Introduction

Clinical data are highly heterogeneous, primarily because real-world data (RWD) are observational and lack the structured format found in controlled environments, such as randomized controlled trials [1]. In addition, the completeness of RWD depends on various clinical settings and individual patient cases [2]. For instance, some patients may have extensive records owing to frequent monitoring, whereas others may have sparse data owing to fewer interactions with healthcare providers.

Two challenges arise from the heterogeneity of clinical data: missing data and informative presence [3]. Missing data are common in clinical datasets, which not only add to heterogeneity but also affect the reliability and accuracy of predictive models [4,5,6]. The rate of missingness in clinical data can vary widely owing to factors such as incomplete patient records, loss of follow-up, or errors in data collection. Understanding the mechanisms underlying this missingness is essential to appropriately address this issue. Missing data mechanisms are typically categorized into three types: missing completely at random (MCAR), missing at random (MAR), and missing not at random (MNAR). MCAR occurs when missingness is entirely random, MAR occurs when missingness depends on observed data, and MNAR occurs when missingness is related to unobserved data itself [7,8].

Another important concept related to missing data and heterogeneity that warrants attention is informative presence, which refers to the idea that the act of observing data points is meaningful; the presence of data can carry significant information about a patient’s condition. However, despite its potential importance, a review by Goldstein et al. that encompassed 107 clinical predictive modeling studies found that none formally investigated the influence of informative presence on predictive models [3].

Given the widespread heterogeneity that can adversely affect the performance of AI predictive models [9,10,11], especially deep learning-based models, it is crucial to understand how these factors impact model performance. Developing robust clinical decision support systems (CDSS) that can reliably predict patient outcomes requires a thorough examination of how data heterogeneity affects these models.

The VitalCare–Major Adverse Event Score (VC-MAES) is a proprietary AI CDSS model that employs a deep learning algorithm to predict clinical deterioration events such as unplanned ICU transfers, cardiac arrests, or deaths in patients in general wards up to 6 h before they occur. VC-MAES requires six essential parameters, including age and five vital signs, to calculate the risk score (MAES), which assesses the risk of clinical deterioration. In addition, if available, the model uses 13 additional parameters, including SpO2, Glasgow Coma Scale (GCS), and laboratory data, to enhance its predictive accuracy.

In this study, we evaluated the performance of the VC-MAES model with varying levels of clinical data availability. Specifically, the effectiveness of the model was assessed when informative data were deliberately omitted by inducing missingness. Additionally, we examined the model performance when missing data were imputed using either mean-based random values drawn from the corresponding patient group or multiple imputation by chained equations (MICE), simulating laboratory values that might have been observed if the physicians had chosen to order those tests. These results were compared to those obtained using the default normal-value imputation method.

## 2. Materials and Methods

### 2.1. Study Setting

This study used real-world data from a prospective observational external validation study conducted at Keimyung University Dongsan Hospital (KUDH) in Daegu, Republic of Korea. Data were collected from patients admitted to six general wards in the Internal Medicine (IM) and Obstetrics and Gynecology (OBGYN) departments between June 2023 and January 2024. Patient monitoring continued until discharge or 30 April 2024, whichever occurred first. The model generated predictions in real-time; however, these predictions were neither disclosed to healthcare providers nor incorporated into clinical decision-making processes.

The study adhered to the ethical guidelines of the Declaration of Helsinki, 1975, was approved by the Institutional Review Board (IRB) of KUDH (IRB No. 2022-12-081), which waived the requirement for informed consent, and was registered with the Clinical Research Information Service (CRIS), operated by the National Institute of Health under the Korea Disease Control and Prevention Agency (CRIS Registration Number: KCT0008466).

### 2.2. Study Population

The study population comprised adult patients (≥19 years) who had complete documentation of basic vital signs—systolic and diastolic blood pressure (SBP and DBP), heart rate (HR), respiratory rate (RR), and temperature. Patients who were transferred directly to the ICU from the emergency department (ED) or operating room were considered to have planned ICU transfers and were excluded from the study.

### 2.3. Data Collection

All relevant patient demographic and clinical information, such as vital signs, code status, surgery start and end times, medication orders, and laboratory results, were obtained from electronic health records (EHR). Additional details regarding ICU admission and discharge, time of death, and CPR initiation and termination were collected.

### 2.4. VC-MAES Model Architecture and Data Handling

VC-MAES is a proprietary predictive model that employs a deep learning algorithm to handle time-series clinical data. This binary classification model, built on a bidirectional long short-term memory (biLSTM) framework, was designed to predict the likelihood of major adverse events in general ward inpatients. It incorporates two types of input data: (1) dynamic features represented as time-series data—sampled hourly to capture vital signs and blood test results, and (2) static features. The dynamic features were processed using the biLSTM network, whereas the static features were managed via fully connected layers. The outputs from both the biLSTM and the fully connected layers were then merged and fed into additional classification layers for final predictions. A schematic diagram of the VC-MAES model architecture is presented in Supplementary Figure S1. Further details of this classification model are provided by Sung et al. [12].

The model was trained using a comprehensive dataset from the Yonsei Severance Hospital in Seoul, Republic of Korea, encompassing over 300,000 hospitalizations across more than 35 medical and surgical specialties from 2013 to 2017. The primary objective of VC-MAES is to predict clinical deterioration events within a six-hour window for patients in medical-surgical wards.

The VC-MAES generates risk scores on a scale of 0–100 based on six core inputs: five standard vital signs, including SBP, DBP, HR, RR, temperature, and patient age. Higher scores indicate an increased likelihood of adverse events occurring within the subsequent 6 h. When 13 additional parameters are available (oxygen saturation, GCS, total bilirubin, lactate, creatinine, platelets, pH, sodium, potassium, hematocrit, white blood cell count, bicarbonate, and C-reactive protein), the model incorporates these parameters to calculate a more comprehensive MAES.

To address missing data, the system employs the last observation carried forward (LOCF) method [13,14], whereby missing values are replaced with the most recently observed values. When no prior data are available, the system defaults to a normal-value imputation method that incorporates standard reference values (Supplementary Table S1).

Owing to institutional practices restricting mental status evaluations to ICU settings, all GCS values were standardized to 15 for the MAES calculations. Additionally, when determining the National Early Warning Score (NEWS) and the Modified Early Warning Score (MEWS), the level of consciousness was consistently recorded as “Alert”, corresponding to an Alert, Verbal, Pain, Unresponsive (AVPU) score of 0.

### 2.5. Statistical Analysis and Model Performance Evaluation

We compared the characteristics of the patients in the control and event groups using chi-square tests for categorical variables and *t*-tests or Wilcoxon rank-sum tests for continuous variables, as appropriate.

Model performance was assessed using the area under the receiver operating characteristic curve (AUROC) and the area under the precision–recall curve (AUPRC) across three distinct scenarios.

In the first scenario, to eliminate the informative presence in the data, we intentionally induced missingness by omitting actual data points, except for the five essential vital signs and age information. We then calculated the AUROC using only vital signs and age and compared the outcomes with those from NEWS and MEWS.

In the second scenario, we reintroduced the available SpO2 and laboratory data to assess how the actual clinical data points influenced the performance of the model.

In the third scenario, to simulate the availability of all 11 laboratory test results, missing values at admission were imputed by randomly drawing values within one standard deviation (SD) of the mean observed values. This approach served as our primary comparison method and was applied separately to the event and non-event groups to more accurately reflect each group’s underlying distribution. To further validate this primary result, we conducted a secondary analysis comparing normal-value imputation with MICE, an advanced method shown in a previous study to outperform mean-based imputation [15].

Furthermore, to investigate whether missing data patterns alone could provide an “informative presence” that enhanced model performance, we developed two experimental models with the same VC-MAES architecture but different input features. One model relied solely on five vital signs (SBP, DBP, HR, RR, and temperature) and age, while the other model incorporated those same vital signs plus a binary lab ordering indicator, denoting whether laboratory tests were ordered, without using the actual laboratory values.

We evaluated and compared the AUROC for each scenario to determine the impact of varying levels of missing data and informative presence on the predictive performance of the model. The DeLong test was used to compare the AUROCs of the predictive models.

## 3. Results

### 3.1. Baseline Characteristics

A total of 6478 initial patient encounters, representing 4846 patients admitted to general wards, were screened. After applying the exclusion criteria, 439 cases were excluded: 423 owing to direct ICU admissions from either the ED or operating room and 16 owing to incomplete scoring data. The final analysis included 6039 cases involving 4447 individuals.

Adverse events (AEs) occurred in 217 patients, representing 3.6% of all cases. These AEs included 102 unplanned ICU transfers, 13 instances of cardiac arrest, and 102 mortalities.

The demographic profile revealed a mean age of 53.10 years, with notable differences between groups. Patients experiencing AEs were significantly older (mean age, 74.04 years), compared to those without complications (mean age, 52.32 years) (*p* < 0.001).

The study population demonstrated a marked sex imbalance, with 78.90% of the patients being female. This skew is attributable to the departmental distribution, as the OBGYN unit accounted for approximately 64% of the study cases, resulting in a predominantly female sample.

Baseline demographic characteristics and vital signs of the event and control groups are summarized in Table 1.

### 3.2. Missing Laboratory Test Frequencies

Table 2 illustrates the differences in the frequency of missing laboratory test results between the non-event and event groups. The non-event group (n = 5822) generally exhibited a higher proportion of missing values across several laboratory measures. For instance, over 80% of the lactate and pH data were missing in the non-event group, compared to only 7.37% and 6.91%, respectively, in the event group. In contrast, the event group (n = 217) had substantially fewer missing values for multiple laboratory parameters, which often approached zero. For the laboratory contribution to the MAES, hematocrit was identified as the most important laboratory feature associated with events, followed by HCO_3_^−^, platelet count, and lactate. A detailed illustration of these feature-importance results is provided in Supplementary Figure S2.

### 3.3. Vital-Signs-Only Performance with Induced Missingness

**Figure 1:**
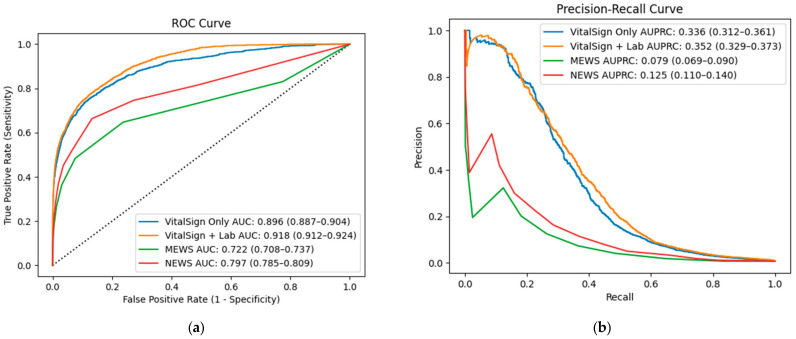
Receiver Operating Characteristic Curves (**a**) and Precision–Recall Curves (**b**) comparing predictive performance among models using vital signs only, vital signs + laboratory data, Modified Early Warning Score (MEWS), and National Early Warning Score (NEWS).

When VC-MAES utilized only vital signs while treating other data as missing and imputing normal values using the model’s default method, it achieved an AUROC of 0.896 (95% CI: 0.887–0.904) and an AUPRC of 0.336 (95% CI: 0.312–0.361). In comparison, the NEWS and MEWS scores had AUROCs of 0.797 (95% CI: 0.785–0.809) and 0.722 (95% CI: 0.708–0.737), respectively, with corresponding AUPRCs of 0.125 (95% CI: 0.110–0.140) and 0.079 (95% CI: 0.069–0.090) for MEWS. These results indicate that VC-MAES outperformed both NEWS and MEWS in terms of both AUROC and AUPRC (Figure 1).

Upon reintroducing the full set of originally collected clinical data, previously treated as missing, the VC-MAES performance improved significantly compared with the vital-signs-only scenario. The AUROC increased from 0.896 to 0.918 (95% CI: 0.912–0.924; DeLong test *p* < 0.001), and the AUPRC increased from 0.336 (95% CI: 0.312–0.361) to 0.352 (95% CI: 0.329–0.373), further enhancing the model’s predictive capabilities.

### 3.4. Performance with Forced Mean-Based Imputation and MICE Imputation

When missing values were imputed using values randomly drawn from within one SD of the mean observed values for the corresponding non-event and event groups, the AUROC decreased to 0.883 (95% CI: 0.875–0.891) compared with 0.918 (95% CI: 0.912–0.924) under the model’s default normal-value imputation method (DeLong test, *p* < 0.001).

A similar trend was observed in the AUPRC, which declined from 0.352 (95% CI: 0.329–0.373) under the default method to 0.307 (95% CI: 0.285–0.329) under forced mean-based imputation (DeLong test, *p* < 0.001).

Furthermore, at a fixed sensitivity of 80%, the specificity under the default imputation method was 85.5%, which decreased to 78.3% under forced mean-based imputation. These results indicate that mean-based random value replacement negatively affects the discriminative ability of a model.

When analyzing the model’s prediction scores, MAES, the average score of the event group was 52.6 with the normal-value imputation, which remained similar at 52.8 with forced mean-based imputation. However, in the non-event group, the average score increased to 10.6 with forced mean-based imputation from 7.7 with the normal-value imputation. The distribution of the average scores in each group for each imputation method is shown in Supplementary Figure S3.

In the secondary analysis using the MICE imputation method, the AUROC declined further to 0.827 (95% CI: 0.824–0.830), compared with both the model’s default normal-value imputation and the forced mean-based imputation method. Supplementary Figure S4 illustrates the AUROC achieved by each method.

**Figure 2:**
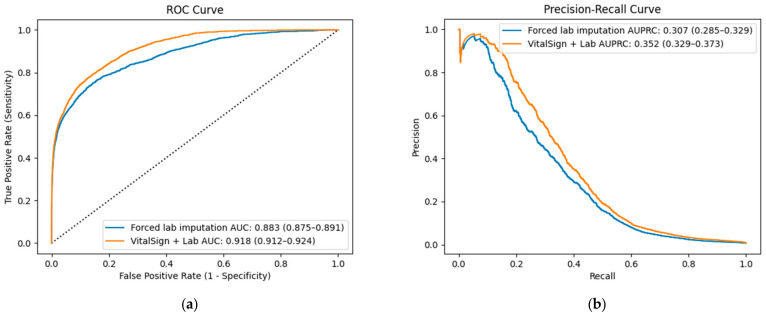
Receiver Operating Characteristic Curves (**a**) and Precision–Recall Curves (**b**) comparing predictive performance among models using vital signs + laboratory data and forced lab imputation.

The baseline laboratory values are presented in Table 3, comparing the raw data without imputation and the forced mean-based imputation approach for both non-event and event groups. The performance of VC-MAES under these two methods is illustrated in Figure 2.

### 3.5. Performance with a Model Incorporating a Lab-Ordering Pattern

When comparing a model that relied solely on five vital signs and age with a second model that also included a binary lab-ordering indicator to denote whether laboratory tests were ordered without using the actual lab values, the latter model demonstrated superior performance. Specifically, the model incorporating the lab-ordering pattern achieved an AUROC of 0.849 (95% CI: 0.846–0.851), compared with 0.831 (95% CI: 0.828–0.834) for the vital-signs-only model (Supplementary Figure S5).

## 4. Discussion

In this study, we evaluated the performance of the VC-MAES model under varying levels of clinical data availability by systematically adjusting the extent of missing data. Even when limited to vital signs and age, the model demonstrated robust predictive performance, outperforming established early warning scores such as NEWS and MEWS. Incorporating additional real-world clinical data, including SpO_2_ and laboratory values, further enhanced predictive accuracy. In contrast, forcing the imputation of missing values through mean-based estimates from both event and non-event groups or MICE reduced performance relative to the model’s default normal-value imputation. This decline underscores that ignoring the underlying reasons why tests were not ordered can obscure clinically significant signals about patient status, suggesting the value of preserving informative presence and emphasizing the importance of carefully handling missing data patterns.

In real-world clinical practice, data availability varies owing to factors such as loss of follow-up, documentation errors, practice patterns, resource constraints, and patient preferences. When data are absent, they are typically considered “missing”. However, this term can be misleading because it suggests that data are required but are simply unavailable. Certain tests may not have been indicated or were deemed unnecessary, thereby illustrating the concept of informative observations. The presence of data provides meaningful information about the patient’s condition and the clinical decision-making process [3].

Our study exemplified this finding. For example, we observed substantial differences in the frequency of missing laboratory tests between the non-event and event groups. Patients who experienced adverse events had far fewer missing laboratory values (e.g., lactate and pH), likely because clinicians suspected potential deterioration and ordered more comprehensive testing. In contrast, patients in the non-event group often lacked these tests, reflecting the clinical judgment that they were stable and did not require further investigation. Such differences support the notion that the absence of data is not merely a void but an informative pattern tied closely to clinical decision-making. This interpretation is further supported by our experiment comparing two models: one using only vital signs and another incorporating both vital signs and a lab-ordering pattern. The model that included the lab-ordering pattern outperformed the vital-signs-only model, despite not using actual laboratory values. This finding underscores the significance of “patterns” in missing or present data for predictive accuracy.

We specifically examined the model’s performance under three scenarios: (1) using only the minimum required data (five vital signs and age), (2) reintroducing originally collected data addressed through normal value imputation, and (3) imputing missing data either by drawing random values from within one SD of the mean observed values of the event and non-event groups or by using MICE to approximate what values might have been if clinicians had chosen to order those tests.

The VC-MAES model demonstrated a strong predictive capability using only vital signs and age, achieving an AUROC of 0.896 and outperforming both NEWS (0.797) and MEWS (0.722). This suggests that even a limited set of essential features can yield effective predictions, possibly because the model’s deep learning architecture captures complex patterns that conventional scoring systems may miss [16,17].

Upon reintroducing the previously withheld clinical data, the performance of the model improved significantly, with the AUROC increasing from 0.896 to 0.918. This substantial enhancement underscores the importance of obtaining comprehensive clinical information. While basic vital signs provide a strong predictive foundation, incorporating additional real-world parameters can improve the ability of the model to identify at-risk patients, which is consistent with previous studies [18,19].

Conversely, forcibly imputing missing data with mean-based values reduced the model’s AUROC from 0.918 to 0.883, indicating that this approach failed to account for the valuable clinical context inherent in missingness patterns [20,21,22]. The observed decline in performance was largely driven by an increase in false-positive rates within the non-event group, which had a significantly higher proportion of missing laboratory data. As a result, these patients were more susceptible to forced laboratory imputation, leading to substantial changes in average MAES scores and their distribution, as noted in Section 3. In the secondary analysis, MICE imputation yielded an AUROC of 0.827, which was lower than the model’s default normal-value imputation. This finding aligns with our primary results using mean-based imputation and further underscores the importance of preserving clinically meaningful data patterns.

Normal-value imputation is designed to mirror a common clinical assumption: if a test was not ordered, it may be because nothing appeared abnormal enough to warrant it, implying that the patient might be “normal” for that parameter. By providing plausible baseline measurements rather than arbitrary replacements, normal-value imputation aligns more closely with clinicians’ decision-making processes [23]. Although no imputation method should serve as the sole basis for clinical decision-making, this clinically informed approach may help preserve the meaningful structure derived from clinical judgment.

Clinicians order or forgo tests based on their professional assessments. The absence of certain measurements often indicates that no additional testing was deemed necessary, suggesting patient stability. In contrast, frequent or specialized testing may signal mounting concern about potential deterioration. This clinical assessment remains a cornerstone of medical practice, delivering substantial value in patient care and diagnosis by ensuring that care is both efficient and effective. Ignoring this and treating missing data merely as random gaps, rather than as meaningful indicators grounded in clinical decision-making, obscures these nuances and diminishes a model’s capacity to accurately reflect patient status [24]. Additionally, from a practical standpoint, our findings suggest that models such as VC-MAES can be implemented effectively without exhaustive data collection or overly aggressive imputation strategies.

This study has the following limitations. First, it was conducted at a single center in South Korea, which may limit the generalizability of the findings to institutions with different patient populations or clinical practices. However, the novelty of this work lies primarily in investigating informative presence in a real-world dataset and comparing various imputation strategies. Additionally, multicenter external validations are currently underway, and the findings from this study will be assessed across broader datasets to strengthen and confirm its applicability in diverse clinical settings.

Second, this study did not assess the impact of varying proportions of missing data or informative presence on model robustness. In clinical environments, the amount and distribution of data can vary significantly, and understanding how these factors affect the model performance is crucial for ensuring generalizability and reliability.

Future studies should explore the generalizability of these findings across different clinical settings and populations. Additionally, developing advanced imputation methods that acknowledge the nonrandom nature of missing data and preserve informative patterns could further improve predictive performance.

## 5. Conclusions

In conclusion, our study demonstrated the substantial influence of missing data and informative presence on the performance of predictive models, such as VC-MAES. The VC-MAES model proved highly effective in predicting clinical deterioration using minimal data and outperformed traditional early warning scores. The incorporation of available laboratory data further enhanced the predictive accuracy of the model, underscoring the value of comprehensive clinical information when available. Importantly, our findings revealed that using actual clinical data with appropriate handling of missing values was superior to forcefully imputing missing data to simulate complete datasets. The pattern of missingness itself contains valuable clinical insights that reflect both clinicians’ decision-making processes and patients’ underlying conditions.

## Figures and Tables

**Table 1:** Baseline demographic characteristics and vital sign values of the non-event and event groups.

Variables	Overall	Non-Event	Event	*p*-Value
Number of patients	6039	5822	217	
Demographics, mean (SD)				
Age, y	53.10 (17.93)	52.32 (17.59)	74.04 (13.69)	<0.001
Sex, n (%)				
Male	1274 (21.09)	1146 (19.68)	128 (58.99)	<0.001
Female	4765 (78.90)	4676 (80.32)	89 (41.01)	<0.001
Department, n (%)				
IM	2177 (36.05)	1970 (33.84)	207 (95.39)	
OBGYN	3862 (63.95)	3852 (66.16)	10 (4.61)	
Height, cm	160.23 (7.55)	160.191 (7.49)	161.117 (9.13)	0.142
Weight, kg	62.87 (13.51)	63.13 (13.48)	55.83 (12.20)	<0.001
Body mass index, kg/m^2^	24.41 (4.56)	24.52 (4.54)	21.46 (4.09)	<0.001
Vital signs, mean (SD)				
Pulse/min	82.34 (14.88)	81.97 (14.60)	92.37 (18.42)	<0.001
Respiratory rate/min	19.64 (2.38)	19.55 (2.13)	22.167 (5.38)	<0.001
Systolic blood pressure, mmHg	124.62 (17.30)	124.50 (17.16)	127.67 (20.74)	0.028
Diastolic blood pressure, mmHg	72.97 (11.94)	72.95 (11.88)	73.40 (13.48)	0.630
Body temperature, °C	36.79 (0.37)	36.78 (0.36)	36.96 (0.55)	<0.001
Saturation point O_2_ (%)	97.42 (2.23)	97.47 (2.05)	96.52 (4.32)	0.001

IM: Internal Medicine, OBGYN: Obstetrics and Gynecology.

**Table 2:** Frequency of missing laboratory results in non-event vs. event groups.

Laboratory, n (%)	Total (n = 6039)	Non-Event (n = 5822)	Event (n = 217)
Total bilirubin	216 (3.58)	216 (3.71)	0 (0.00)
Lactate	4866 (80.58)	4850 (83.30)	16 (7.37)
pH	4829 (79.96)	4814 (82.69)	15 (6.91)
Sodium	157 (2.60)	157 (2.70)	0 (0.00)
Potassium	157 (2.60)	157 (2.70)	0 (0.00)
Creatinine	216 (3.58)	216 (3.71)	0 (0.00)
Hematocrit	23 (0.38)	23 (0.40)	0 (0.00)
White blood cell count	23 (0.38)	23 (0.40)	0 (0.00)
HCO_3_^−^	4830 (79.98)	4814 (82.69)	16 (7.37)
Platelet	23 (0.38)	23 (0.40)	0 (0.00)
C-reactive protein	2393 (39.63)	2391 (41.07)	2 (0.92)

**Table 3:** Comparison of baseline laboratory results in non-event vs. event groups, contrasting raw data (no imputation) and forced mean-based imputation.

Laboratory (Mean [SD])	Non-Event (Raw Data)	Event (Raw Data)	*p*-Value	Non-Event (Mean-Based)	Event (Mean-Based)	*p*-Value
Total bilirubin (mg/dL; ref. 0.1–1.0)	0.46 (0.43)	0.66 (0.45)	<0.001	0.48 (0.35)	0.59 (0.33)	<0.001
Lactate (mmol/L; ref. 0.6–2.3)	1.50 (1.30)	2.09 (1.94)	<0.001	1.57 (1.16)	1.84 (1.42)	<0.001
pH (ref. 7.35–7.45)	7.42 (0.08)	7.39 (0.11)	0.014	7.42 (0.08)	7.40 (0.10)	0.015
Sodium (mmol/L; ref. 135–145)	136.48 (3.71)	134.52 (6.46)	<0.001	136.84 (3.57)	134.83 (5.67)	<0.001
Potassium (mmol/L; ref. 3.6–5.2)	4.26 (0.48)	4.40 (0.82)	0.012	4.27 (0.46)	4.45 (0.73)	<0.001
Creatinine (mg/dL; ref. 0.8–1.3)	0.92 (0.95)	1.93 (2.26)	<0.001	0.94 (0.89)	1.30 (1.82)	<0.001
Hematocrit (%; ref. 38.8–50)	35.66 (5.174)	34.55 (6.61)	0.015	36.21 (5.20)	35.19 (6.60)	0.005
White blood cell count (10^3^/µL; ref. 3.5–10.5)	9.05 (4.48)	11.554 (7.54)	<0.001	8.15 (3.79)	10.12 (6.43)	<0.001
HCO_3_^−^ (mmol/L; ref. 22–29)	24.10 (4.91)	23.59 (6.03)	0.272	24.17 (4.82)	23.84 (6.39)	0.332
Platelets (10^3^/µL; ref. 150–450)	233.86 (81.25)	232.76 (123.52)	0.896	244.19 (81.56)	240.44 (119.58)	0.514
C-reactive protein (mg/dL; ref. 0.0–0.8)	2.80 (5.54)	9.38 (9.00)	<0.001	3.53 (4.08)	7.19 (7.28)	<0.001

The raw data columns include only patients with recorded laboratory measurements, whereas the mean-based imputation columns encompass all patients, with any missing values replaced by random values drawn from within one standard deviation of the observed mean. ref., reference ranges.

## Data Availability

The data used in the current study can be obtained from the corresponding author upon reasonable request.

## Supplementary Materials

The following supporting information can be downloaded at: https://www.mdpi.com/article/10.3390/jcm14072213/s1, Supplementary Table S1. Normal values for missing value imputation; Supplementary Figure S1. Schematic diagram of the VC-MAES model architecture; Supplementary Figure S2. Top 10 most important features; Supplementary Figure S3. Distribution of the average scores in each group for each imputation method (original normal-value imputation vs. forced mean-based imputation); Supplementary Figure S4. Receiver Operating Characteristic Curves comparing predictive performance among models using vital signs + laboratory data, forced lab imputation (mean-based), and multiple imputation by chained equations (MICE); Supplementary Figure S5. Receiver Operating Characteristic Curves comparing predictive performance among models using vital signs only and binary vital signs and lab pattern.

## References

[1] Liu F., Panagiotakos D. (2022). Real-world data: A brief review of the methods, applications, challenges and opportunities. BMC Med. Res. Methodol..

[2] Mendelsohn A.B., Dreyer N.A., Mattox P.W., Su Z., Swenson A., Li R., Turner J.R., Velentgas P. (2015). Characterization of Missing Data in Clinical Registry Studies. Ther. Innov. Regul. Sci..

[3] Goldstein B.A., Navar A.M., Pencina M.J., Ioannidis J.P.A. (2017). Opportunities and challenges in developing risk prediction models with electronic health records data: A systematic review. J. Am. Med. Inform. Assoc..

[4] Getzen E., Ungar L., Mowery D., Jiang X., Long Q. (2023). Mining for equitable health: Assessing the impact of missing data in electronic health records. J. Biomed. Inform..

[5] Ayilara O.F., Zhang L., Sajobi T.T., Sawatzky R., Bohm E., Lix L.M. (2019). Impact of missing data on bias and precision when estimating change in patient-reported outcomes from a clinical registry. Health Qual. Life Outcomes.

[6] Yang D.X., Khera R., Miccio J.A., Jairam V., Chang E., Yu J.B., Park H.S., Krumholz H.M., Aneja S. (2021). Prevalence of missing data in the national cancer database and association with overall survival. JAMA Netw. Open.

[7] Little R.J., D’Agostino R., Cohen M.L., Dickersin K., Emerson S.S., Farrar J.T., Frangakis C., Hogan J.W., Molenberghs G., Murphy S.A. (2012). The prevention and treatment of missing data in clinical trials. N. Eng. J. Med..

[8] Lee K.J., Carlin J.B., Simpson J.A., Moreno-Betancur M. (2023). Assumptions and Analysis Planning in Studies with Missing Data in Multiple Variables: Moving beyond the MCAR/MAR/MNAR Classification. Int. J. Epidemiol..

[9] Babar M., Qureshi B., Koubaa A. (2024). Investigating the impact of data heterogeneity on the performance of federated learning algorithm using medical imaging. PLoS ONE.

[10] Shadbahr T., Roberts M., Stanczuk J., Gilbey J., Teare P., Dittmer S., Thorpe M., Torné R.V., Sala E., Lió P. (2023). The impact of imputation quality on machine learning classifiers for datasets with missing values. Commun. Med..

[11] Bhatt N., Bhatt N., Prajapati P., Sorathiya V., Alshathri S., El-Shafai W. (2024). A Data-Centric Approach to Improve Performance of Deep Learning Models. Sci. Rep..

[12] Sung M., Hahn S., Han C.H., Lee J.M., Lee J., Yoo J., Heo J., Kim Y.S., Chung K.S. (2021). Event Prediction Model Considering Time and Input Error Using Electronic Medical Records in the Intensive Care Unit: Retrospective Study. JMIR Med. Inform..

[13] Lewis M., Elad G., Beladev M., Maor G., Radinsky K., Hermann D., Litani Y., Geller T., Pines J.M., Shapiro N.L. (2021). Comparison of deep learning with traditional models to predict preventable acute care use and spending among heart failure patients. Sci. Rep..

[14] Zhu X. (2014). Comparison of Four Methods for Handing Missing Data in Longitudinal Data Analysis through a Simulation Study. Open J. Stat..

[15] Mera-Gaona M., Neumann U., Vargas-Canas R., López D.M. (2021). Evaluating the Impact of Multivariate Imputation by MICE in Feature Selection. PLoS ONE.

[16] Shao J., Zhong B. (2003). Last Observation Carry-Forward and Last Observation Analysis. Stat. Med..

[17] Reardon P.M., Parimbelli E., Wilk S., Michalowski W., Murphy K., Shen J., Herritt B., Gershkovich B., Tanuseputro P., Kyeremanteng K. (2021). Machine learning–based early warning systems for clinical deterioration: Systematic scoping review. J. Med. Internet Res..

[18] Reardon P.M., Parimbelli E., Wilk S., Michalowski W., Murphy K., Shen J., Herritt B., Gershkovich B., Tanuseputro P., Kyeremanteng K. (2019). Incorporating laboratory values into a machine learning model improves in-hospital mortality predictions after rapid response team call. Crit. Care Explor..

[19] Boulitsakis Logothetis S., Green D., Holland M., Al Moubayed N. (2023). Predicting acute clinical deterioration with interpretable machine learning to support emergency care decision making. Sci. Rep..

[20] Tsiampalis T., Panagiotakos D.B. (2020). Missing-data analysis: Socio-demographic, clinical and lifestyle determinants of low response rate on self-reported psychological and nutrition related multi- item instruments in the context of the ATTICA epidemiological study. BMC Med. Res. Methodol..

[21] Marino M., Lucas J., Latour E., Heintzman J.D. (2021). Missing data in primary care research: Importance, implications and approaches. Fam. Pract..

[22] Karakaya J., Karabulut E., Yucel R.M. (2015). Sensitivity to Imputation Models and Assumptions in Receiver Operating Characteristic Analysis with Incomplete Data. J. Stat. Comput. Simul..

[23] Luo Y. (2021). Evaluating the State of the Art in Missing Data Imputation for Clinical Data. Brief. Bioinform..

[24] Wang H., Tang J., Wu M., Wang X., Zhang T. (2022). Application of Machine Learning Missing Data Imputation Techniques in Clinical Decision Making: Taking the Discharge Assessment of Patients with Spontaneous Supratentorial Intracerebral Hemorrhage as an Example. BMC Med. Inform. Decis. Mak..

