# Supplementary material for: Preserving Informative Presence: How Missing Data and Imputation Strategies Affect the Performance of an AI-Based Early Warning Score
Source: J Clin Med. 2025 Mar 24;14(7):2213. doi: 10.3390/jcm14072213 (PMC11989256; doi:10.3390/jcm14072213)

Supplementary Table S1. Normal values for missing value imputation

Feature	Imputation normal value
SpO2 (%)	100
GCS	15
Total bilirubin (mg/dL)	0.6
Lactate (mmol/L)	0.7
Creatinine (mg/dL)	0.8
Platelets (10^3/μL)	300
pH	7.4
Sodium (mmol/L)	140
Potassium (mmol/L)	4.2
Hematocrit (%)	45
White blood cell count (10^3/μL)	7
HCO_3^- (mmol/L)	24
C-reactive protein (mg/dL)	1.5

Supplementary Figure S1. Schematic diagram of the VC-MAES model architecture

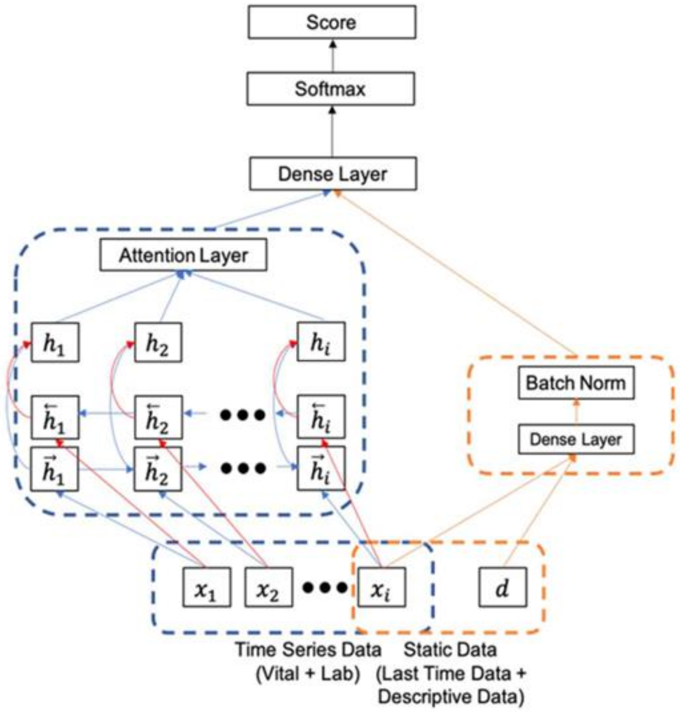

Supplementary Figure S2. Top 10 most important features

(a) Mean absolute SHAP values, indicating each feature's overall contribution to the model's predictions

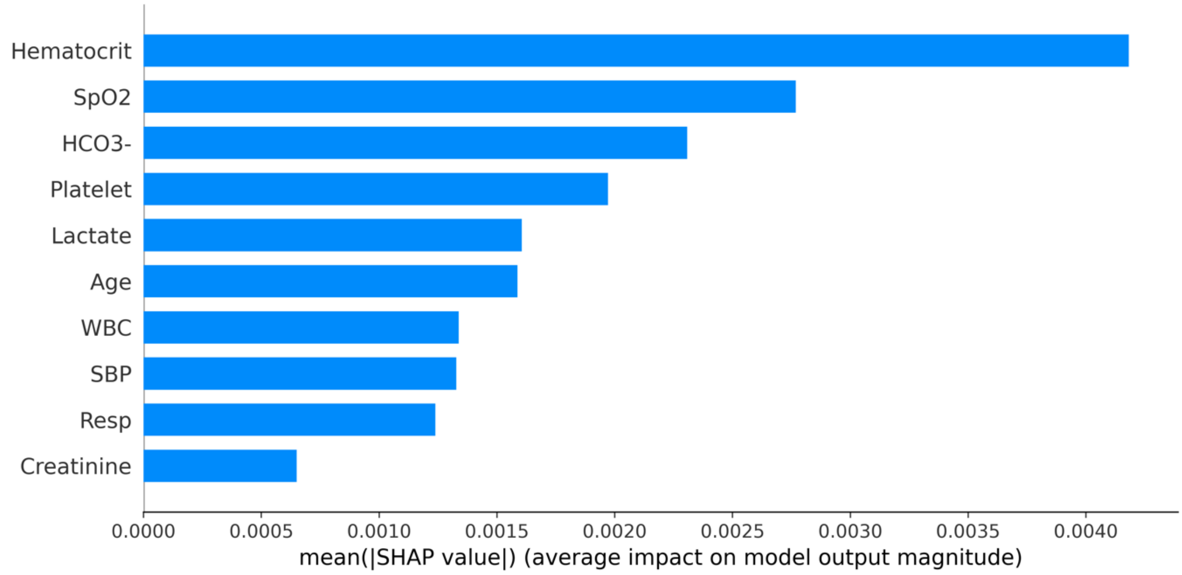

(b) SHAP value beeswarm plot, illustrating how each feature influences the prediction positively or negatively. Dots are colored by feature values, ranging from low (blue) to high (red)

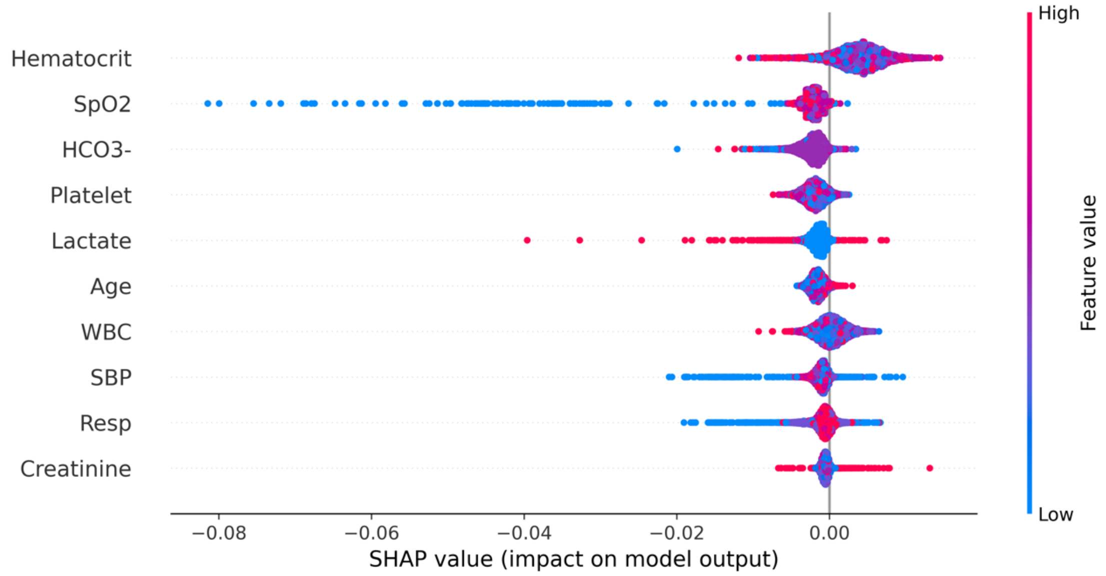

Supplementary Figure S3. Distribution of the average scores in each group for each imputation method (original normal-value imputation vs. forced mean-based imputation)

(a) Non-event group

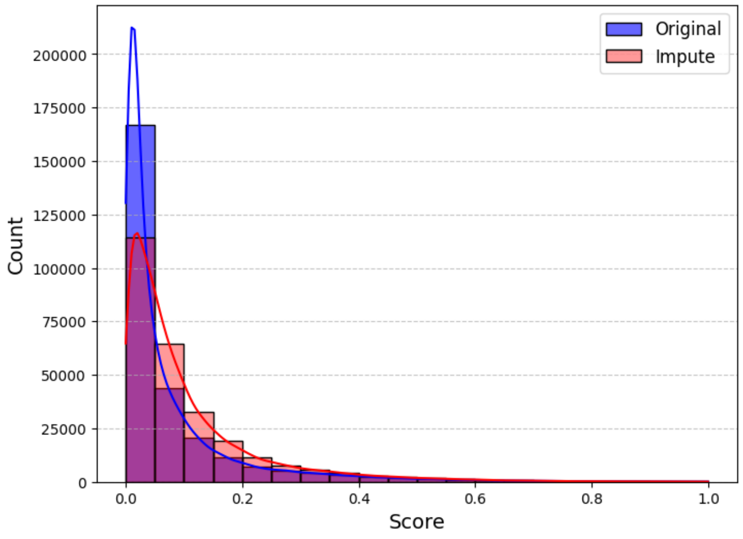

(b) Event group

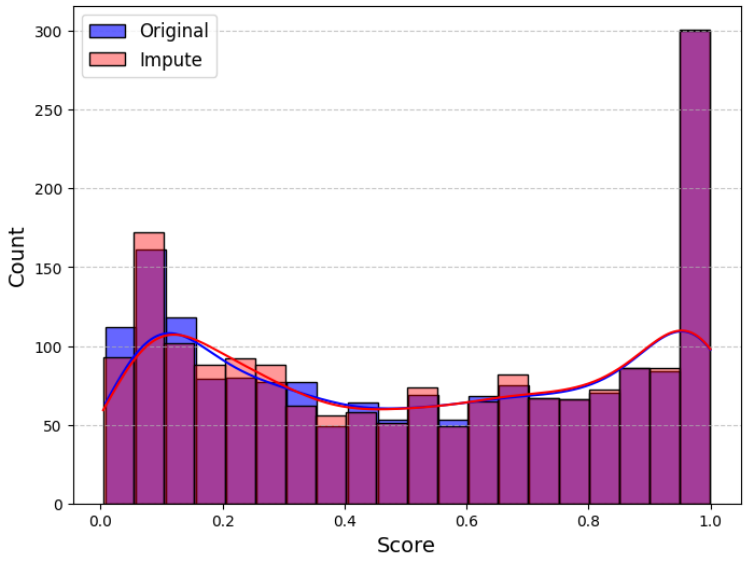

Supplementary Figure S4. Receiver Operating Characteristic Curves comparing predictive performance among models using vital signs + laboratory data, forced lab imputation (mean-based), and multiple imputation by chained equations (MICE)

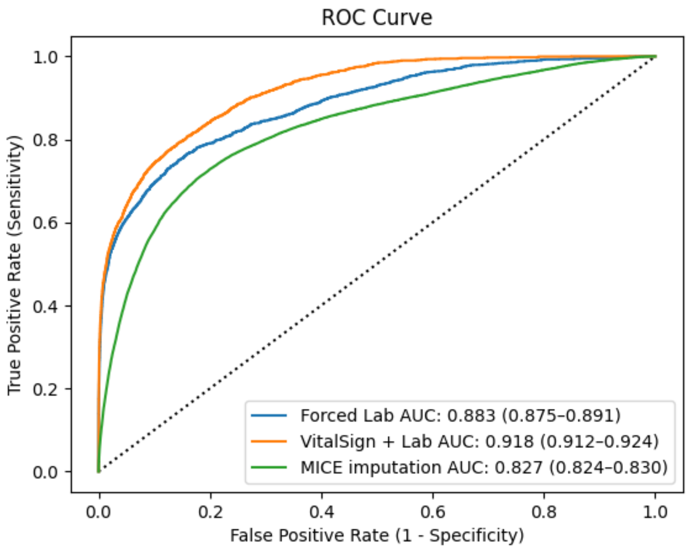

Supplementary Figure S5. Receiver Operating Characteristic Curves comparing predictive performance among models using vital signs only and binary vital signs and lab pattern

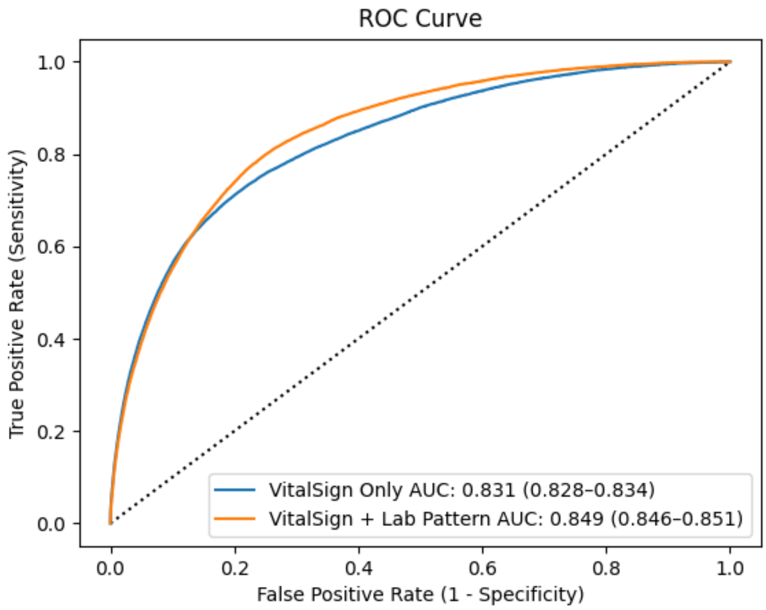

Supplement: Supplementary file 1 [file jcm-14-02213-s001.zip › jcm-3422740-supplementary.pdf]
